# *Tuber magnatum* Picco: the challenge to identify ascoma-associated bacteria as markers for geographic traceability

**DOI:** 10.3389/fmicb.2023.1142214

**Published:** 2023-05-16

**Authors:** Antonio Bucci, Pamela Monaco, Gino Naclerio

**Affiliations:** Department of Biosciences and Territory, University of Molise, Pesche, IS, Italy

**Keywords:** ectomycorrhizal fungi, truffle, ascomata, microbial communities, truffle traceability

## 1. Introduction

Fungi of the genus *Tuber*, also defined as “true truffles,” are ascomycetes belonging to the *Pezizales* order, which grow in symbiosis with the roots of several trees and shrubs (Mello et al., 2006, 2017; Iotti et al., 2016). This genus comprises more than 180 species distributed across Europe, North America, South East Asia and limited parts of Africa and South America (Bonito et al., 2010; Vita et al., 2015; Benucci and Bonito, 2016; Ye et al., 2018; Monaco et al., 2021a). In addition to their ecological role, some truffle species are of considerable economic and commercial importance (Monaco et al., 2022). *Tuber magnatum* Picco, often known as the “Italian white truffle,” is the most expensive of all gourmet truffle species, with a retail price of thousands of euros per kilogram and a trade volume of around 0.9 billion euros per year (Graziosi et al., 2022; Monaco et al., 2022). High truffle prices have led to several forms of adulteration (Hamzić Gregorčič et al., 2020), including the addition of lower-value truffle species (e.g., *T. borchii*) in place of *T. magnatum* (Oliach et al., 2021; Tejedor-Calvo et al., 2023). In addition to this, since the cost of *T. magnatum* can vary depending on its provenience, even at a local/regional scale (Vita et al., 2020; Sillo et al., 2022), it is likely that ascomata collected from less renowned territories can be sold at the same price of truffles of the same species with a higher market value. Moreover, the lack of clear regulations on the manufacturing and labeling of truffled products implies that, regardless of the *Tuber* species used in these products or the presence of flavoring substances, the denomination “truffle”/“truffled” and the images of the most valuable truffle species can be present in any label (Tejedor-Calvo et al., 2023). For instance, a recent study conducted in Spain showed that only 20% of truffled products were properly labeled (Tejedor-Calvo et al., 2023). This “regulatory gap” creates confusion to consumers, depreciates this highly prized product and has a negative impact on producers (Tejedor-Calvo et al., 2023).

Despite several papers have been published on the ecology, genetics and cultivation of truffles, less studies have focused on both the validation of truffle authenticity (Hamzić Gregorčič et al., 2020) and the possibility to differentiate *T. magnatum* on the basis of its origin.

Given the variation in price in relation to its geographic origin (Vita et al., 2020; Sillo et al., 2022), in our opinion, it is of great importance to identify specific markers of *T. magnatum* provenience. This could allow the development of a reliable tracking system with the dual objectives (1) to prevent exposure to fraud in trade and protect consumers, and (2) to identify unique features of truffles from different regions. Ascoma-associated bacteria appear to be promising candidates for *T. magnatum* geographic traceability (Monaco et al., 2021a; Niimi et al., 2021a). In fact, despite information available on bacteria associated with *T. magnatum* ascomata is still limited, there is some evidence that peculiar bacterial taxa can be linked to *T. magnatum* fruiting body provenience. We believe more research should investigate the bacterial communities associated with *T. magnatum*, with the purpose of detecting and identifying microorganisms that could be used as markers of fruiting body origin. The availability of high-throughput sequencing technologies that can be applied to large-scale investigations of *T. magnatum* populations could help researchers to identify these biomarkers, in order to develop easy, rapid, and cheap protocols for their detection.

## 2. Ascoma-associated microbial communities

Complex microbial communities of bacteria, yeasts, filamentous fungi, and viruses are associated with truffles and, in the last few years, a combination of culture-dependent and independent methods has been employed to unravel their composition (Stielow and Menzel, 2010; Splivallo et al., 2015, 2019; Vahdatzadeh et al., 2015, 2019; Benucci and Bonito, 2016; Ratti et al., 2016). Bacteria can heavily colonize truffle inner tissues (gleba) and the surface (peridium), reaching a density from millions to billions of cells per gram (dry weight) (Reale et al., 2009; Splivallo et al., 2015, 2019; Vahdatzadeh et al., 2015, 2019). A specific recruitment of selected genera from the soil microbial communities occurs before the differentiation of ascocarpic tissues, when the primordium is directly in contact with soil (Antony-Babu et al., 2014; Vita et al., 2020; Monaco et al., 2022). After the differentiation of the peridium, which remains in contact with ground throughout the ascoma development, bacteria remain trapped in the gleba (Antony-Babu et al., 2014; Monaco et al., 2020a; Vita et al., 2020). Bacteria can play a key role in the complex biological processes of signaling and nutrient exchanges involving hyphae, ectomycorrhizas, and ascocarps (Barbieri et al., 2016). They produce biostimulants (phytohormones and specific amino acids), participate in the development and maturation of truffle ascomata, improve fungal nutrition, and are involved in spore germination, opening of asci and ascospore release through their enzymatic activities (Mello et al., 2010; Pavić et al., 2011, 2013; Antony-Babu et al., 2014; Amicucci et al., 2018). Some bacteria are able to produce antimicrobial substances that inhibit the growth of pathogens and contaminating fungi. In addition, microorganisms contribute also to the truffle aroma by synthesizing sulphur volatile compounds that not only determine the organoleptic properties of fruiting bodies, but also attract mammals, favoring the dissemination of spores (Splivallo et al., 2011, 2015; Splivallo and Ebeler, 2015).

The composition of the ascoma-associated microbial communities can be influenced by many different factors such as the ascoma maturation degree, the storage period, the collection site, the harvesting season, and the environmental conditions (Vahdatzadeh et al., 2015; Monaco et al., 2021a; Niimi et al., 2021a; Sillo et al., 2022). Only a few studies have characterized microbial communities associated with *T. magnatum*, and most of them have focused on bacteria (Monaco et al., 2021a; Niimi et al., 2021a,b; Marozzi et al., 2022). These studies suggest that there is a predominance of α-*Proteobacteria* and, in particular, of *Bradyrhizobium* species, regardless of fruit body provenience and maturation degree. Fruiting body-associated communities are characterized by a lower diversity compared to the surrounding bulk soil, and a further reduction emerged within the gleba (Sillo et al., 2022). A convergence on few selected taxa (mainly represented by *Mortierella* for fungi, *Bradyrhizobium, Rhizobium, Pseudomonas, Ensifer, Polaromonas, Pedobacter, Chitinophaga*, and *Phyllobacterium* for bacteria), which constitute the “core microbiota” of *T. magnatum* ascomata (Monaco et al., 2021a; Niimi et al., 2021a; Marozzi et al., 2022), has been detected, although factors behind this selection have not yet been fully elucidated (Vahdatzadeh et al., 2015). It is likely that the reduction of diversity and the exclusive presence of certain microbial groups inside the ascomata may be determined by a selective pressure on microorganisms related to specific potential functions and the evolutionary adaptation to the genus *Tuber* of some bacterial taxa (Marozzi et al., 2022; Sillo et al., 2022), testifying to their crucial role in truffle ecology and life cycle. On the other hand, “variable taxa”, “non-fixed” bacterial taxa that underlie specific differences observed among ascoma-microbial communities, can be associated with the “core microbiota” of *T. magnatum* (Monaco et al., 2021a, 2022). In our opinion, this is of upmost importance in the context of this article, since these “variable taxa” may represent microbial markers for geographic traceability.

## 3. Discussion

The several forms of adulteration of truffles and truffle containing products make necessary the identification of appropriate markers and the acquisition of reliable detection methods to prevent issues pertaining to misidentification and fraudulent practices. *Tuber* spp. are identified by truffle hunters by considering fruit body odor, size and shape of spores and asci, spore wall ornamentation, and structure of the peridium and gleba. However, this kind of truffle recognition remains unreliable (Mello et al., 2006; Strojnik et al., 2020). Misidentification is especially problematic for species with similar morphological features, and therefore less valuable truffles can be (un)intentionally traded as high-quality species such as *T. magnatum*. In addition, *T. magnatum* ascomata with different geographic provenience could be sold on the market as the most expensive “Tartufo bianco di Alba”.

Therefore, as previously stated, these authors are convinced that scientific research should be more oriented in detecting and identifying specific markers for truffle geographic traceability. Until now most of the attention has been paid to the analysis of intraspecific genetic variability (Mello et al., 2005; Monaco et al., 2021a), population genetic structure (Rubini et al., 2005; Belfiori et al., 2020), antioxidant compounds (Vita et al., 2018), transcriptomic, proteomic, and volatilomic profiles (Vita et al., 2020), as well as morphological traits, such as peridium thickness (Monaco et al., 2021b).

On the other hand, the characterization of microorganisms associated with truffle ascomata is a more recent research field. Although there is still little scientific evidence, bacteria seem to be promising candidates for tracing the geographic origin of truffles (Monaco et al., 2021a; Niimi et al., 2021a).

Niimi et al. (2021a), for example, analyzed bacterial communities associated with *T. magnatum* populations from different European countries (Croatia, Hungary, Italy, and Serbia) and despite finding a highly variable microbiota, with wide diversity, among fruiting bodies, no matter their provenience, they also detected an OTU belonging to the genus *Pseudomonas* as a potential marker of geographic origin of some Hungarian *T. magnatum* ascomata. Also Monaco et al. (2021a) described that, besides a “core microbiota” shared among ascocarps collected in Central-Southern Italy (Molise region), “non-fixed” bacterial taxa can complete the composition of the truffle microbiota, contributing to determine specific differences between and within the analyzed *T. magnatum* populations. Even though several environmental factors, such as temperature, humidity, soil properties, microclimatic conditions, and snow cover (Monaco et al., 2020b), significantly affect the structure of microbial communities and, even little variations can determine important changes in the truffle microbiota, we hypothesize that potential markers of *T. magnatum* geographic origin could be among these “variable” bacteria.

Despite these first evidences, to date, we are still far from being able to affirm that effective bacterial markers of truffle origin exist, since there are still some difficulties and limitations to overcome, mainly related to the reduced number of studies carried out in this direction and also to the small-scale heterogeneity in truffle microbiota composition, which makes more difficult their detection. In fact, an ideal biomarker should have these requirements: (1) to be almost constantly present in truffles coming from a particular geographical area and absent in fruit bodies with a diverse provenience or, at least, show significantly different abundance values and (2) to be easily detectable through effective, low-cost, and rapid analyses. Therefore, the main challenge of scientific community should be to step up efforts to search for valid biomarkers of origin. The use of next-generation sequencing (NGS) technologies and the subsequent comparison of *T. magnatum* associated-bacterial communities on a full scale could help to achieve this goal, by allowing the identification of those microorganisms on which to focus attention.

The identification of bacterial markers and, in future perspective, the development of a reliable tracking system could contribute to prevent the risk of exposure to fraud in trade for consumers, but also to promote the development of the collection territories.

In fact, the definition of specific markers of *T. magnatum* origin, together with a comprehensive characterization of its organoleptic properties, could contribute to identify unique features of truffles from different regions and to the conservation and promotion of this resource of utmost importance for some local economies. For example, the Molise region, in Central-Southern Italy, has always received less scientific attention compared to other territories, but represents one of the richest areas in Italy for truffle production (especially of the valuable *T. magnatum*), contributing about 40% to the national truffle production and hosting the highest percentage of truffle hunters operating in Italy (Monaco et al., 2020a, 2021a). This small region shows high socio-economic marginality (Mastronardi et al., 2020) and could exploit this resource, if properly valued, to develop food tourism, attract visitors, and stimulate its economy, which presents developmental decay (Mastronardi et al., 2020), through an appropriate truffle placement on the market.

Developing highly practical and extensively applied traceability technologies for agricultural products has become one of the essential research focuses across many nations. Molecular biology techniques, which detect organisms' genetic material, have advantages in specificity, accuracy, and reproducibility and have been used to construct honey, seafood, and plant traceability systems (Liu et al., 2023). In the case of *Tuber magnatum*, the identification of geographically specific bacterial markers could allow the discrimination of ascomata with different origins through the development of easy, rapid and cheap PCR protocols for specific RNA or DNA sequences.

## Author contributions

Conceptualization and supervision: AB. Writing—original draft preparation: AB and GN. Writing-review and editing: AB, PM, and GN. All authors have read and approved the submitted version.
